# Green synthesis of zinc oxide nanoparticles using aqueous extract of shilajit and their anticancer activity against HeLa cells

**DOI:** 10.1038/s41598-024-52217-x

**Published:** 2024-01-25

**Authors:** Parthasarathi Perumal, Nazeer Ahamed Sathakkathulla, Kalaivani Kumaran, Ramaladevi Ravikumar, Justin Jayaraj Selvaraj, Vijayakumar Nagendran, Mariappan Gurusamy, Naazneen Shaik, Senthilkumar Gnanavadivel Prabhakaran, Vinothkumar Suruli Palanichamy, Vellaichamy Ganesan, Purushoth Prabhu Thiraviam, Seshan Gunalan, Suresh Rathinasamy

**Affiliations:** 1 Department of Molecular and Cell Biology Lab, Greensmed Labs, Thoraipakkam, Chennai, 600097 India; 2 Department of Pharmaceutical Chemistry, EGS Pillay College of Pharmacy, Nagapattinam, 611002 India; 3 Department of Pharmaceutics, St. Mary’s College of Pharmacy, Secunderabad, Telangana 500025 India; 4 Department of Pharmaceutical Chemistry, Faculty of Pharmacy, Bharath Institute of Higher Education and Research (BIHER), Chennai, 600044 India; 5 Department of Pharmaceutical Chemistry, Pannai College of Pharmacy (Affiliated to the Tamil Nadu Dr. M.G.R. Medical University, Chennai), Dindigul, 624005 India; 6 Department of Pharmacognosy, C. L. Baid Metha College of Pharmacy, Chennai 600096, India; 7 Centre of Advanced Study in Crystallography and Biophysics, Guindy Campus, University of Madras, Chennai, 600025 India

**Keywords:** Cancer, Nanoscience and technology

## Abstract

In the present study, ZnO nanoparticles have been synthesized using an aqueous extract of shilajit. The nanoparticles were characterized using different techniques such as UV (ultraviolet–visible spectrophotometer), FTIR (Fourier transform infrared), XRD (X-ray diffraction), particle size analysis, SEM (scanning electron microscope) and EDAX (Energy-dispersive X-ray) analysis. The UV absorption peak at 422.40 nm was observed for ZnO nanoparticles. SEM analysis showed the shape of nanoparticles to be spherical, FTIR spectrum confirmed the presence of zinc atoms, particle size analysis showed the nanoparticle size, EDAX confirmed the purity of ZnO nanoparticles whereas XRD pattern similar to that of JCPDS card for ZnO confirmed the presence of pure ZnO nanoparticles. The in vitro anticancer activity of ZnO nanoparticles against the HeLa cell line showed the IC_50_ value of 38.60 μg/mL compared to reference standard cisplatin. This finding confirms that ZnO nanoparticles from shilajit extract have potent cytotoxic effect on human cervical cancer cell lines.

## Introduction

Cancer is a serious burden on human health and is the second leading cause of death around the world. According to the World Health Organization (WHO) estimated that cancer-related deaths are more common in developed countries than in developing countries. Cervical cancer develops in a woman's cervix and is the fourth most common cancer type in women worldwide. In 2020, 604,000 women were diagnosed with cervical cancer worldwide and 342,000 died as a result of the disease^1^. Cancer is typically treated by conventional therapies such as chemotherapy, radiotherapy, and surgery. Although all these therapies are very effective in killing, cancer cells but also result in many serious side effects.

Recently, nanotechnology-based nanosystems, with high biocompatibility, easy surface functionalization, cancer targeting and drug delivery capacity, have demonstrated the potential to overcome these side effects. In recent times, nanotechnology has gained global attention due to its wide-ranging applications in diverse fields, including optoelectronics, biomedical sciences, mechanics, chemical industry, space industries, catalysis, and drug-gene delivery^2–4^. Green nanotechnology is an emerging field in the development of environmentally friendly nano-products and their utilization to achieve sustainable development^5^. The adoption of green synthetic methods has gained significant attention due to the growing demand for cost-effective, clean, non-toxic, biocompatible, and eco-friendly approaches that promote a safe environment.

Unlike other synthetic methods, the biosynthetic route eliminates the use of toxic chemicals, high energy requirements, and the need for elevated pressures and temperatures^6–10^. The biological resources employed in this eco-friendly approach include plant extracts, bacteria, fungi, cyanobacteria, diatoms, seaweed, and enzymes for the synthesis of metal nanoparticles. Green synthesis techniques leverage natural agents such as plant extracts, sugars, vitamins, biodegradable polymers, and microbes, which act as both reductants and capping agents. Moreover, this approach minimizes the use of inorganic materials and primarily utilizes metal nanoparticles (NPs), metal oxides, and salts for nanoparticle fabrication^11–15^. Green synthesis offers several advantages over alternative synthetic methods, including simplicity, cost-effectiveness, reproducibility, and relatively higher stability^16–19^. Extensive research has been conducted on the use of naturally available resources to synthesize various metal nanoparticles, such as gold, silver, zinc, copper, titanium oxide, platinum, magnetite, and nickel^20–23^. The different parts of the plant are used in Indian traditional medicine for the treatment of painful muscular spasm, dysentery, fever, rheumatism, asthma and as an expectorant and purgative^24–28^. The starting materials of the metal nanomaterials are divalent and trivalent metal ions. There are different methods for the preparation of metal nanoparticles like chemical or photochemical methods. By using reducing agents, the metal ions are reduced to the metal nanoparticles. These have a high surface area and have the good adsorption ability of small molecules. They are widely used in different research areas, environmental and bioimaging studies. Not only a single nanoparticle but also the mixing of two or more nanoparticles with the size control can also be achieved. The plant extracts act as reducing and stabilizing agents for biosynthesis. Bio-reduction involves reducing metal ions or metal oxides to zero valence metal NPs with the help of phytochemicals like tannins, polysaccharides, polyphenolic compounds, vitamins and amino acids^29,30^. Among different nanomaterials, no nanoparticles are considered to be safe both in vitro as well as in vivo. Due to its highly biocompatible and biodegradable nature, zinc oxide (ZnO) nanoparticles can be selected as potent nano-platforms for cancer treatment^31–34^. The use of ZnO nanoparticles in cancer treatment has grabbed the interest of researchers and currently, a lot of studies have been published demonstrating the anti-cancerous activity of ZnO nanoparticles^35–37^.

Shilajit, also known as Asphaltum, Black Bitumen, Silaras, salajit, and shilajatu, is a blackish-brown powder or an exudate from high mountain rocks especially in the Himalayas. Shilajit, sometimes mentioned as a mineral tar or resin, is a highly viscous substance readily soluble in water. Though it is now obtained from many other countries, Shilajit was traditionally sourced in India and Tibet. The health benefits of shilajit differ from region to region, depending on the place from which it is extracted. Shilajit contains fulvic acid, humic acid, 3-hydroxydibenzo-α-pyrone, hippuric acid along with a high phenolic residue^38–40^ that has great potential uses in certain diseases due to its various properties, including antioxidant, anti-inflammatory, anti-ulcerogenic, anti-fungal, anti-diabetic, anxiolytic, antiallergic, analgesic, cognitive memory enhancer, and immunomodulatory properties^41,42^. Shilajit also possesses the ability to interact positively with other drugs and acts as a neuroprotective agent against cognitive disorders. Based on this literature, the current research was undertaken to synthesize shilajit-based ZnO nanoparticles and screened their effect on cervical cancer cell lines. To the best of our knowledge, a biological approach using shilajit extract has been used for the first time as a reducing material as well as a surface stabilizing agent for the synthesis of spherical-shaped ZnO-NPs.

## Results and discussion

### UV analysis of ZnO NPs

**Figure 1:**
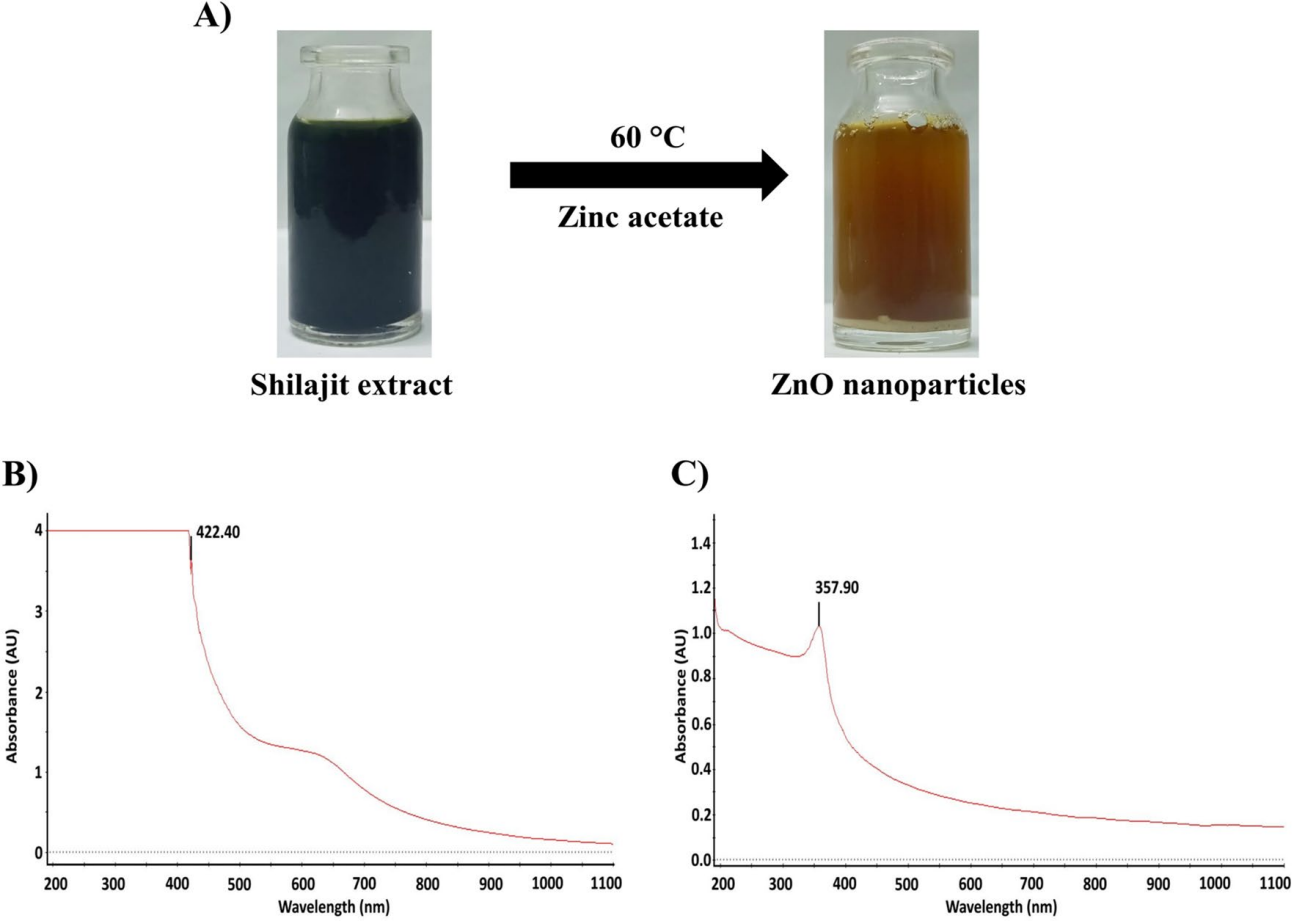
(**A**) Synthesis of ZnO nanoparticles. (**B**) UV–visible absorption spectrum of shilajit extract. (**C**) UV–visible absorption spectrum of ZnO nanoparticles from shilajit extract.

The synthesized ZnO nanoparticles were systematically characterized and screened for anticancer activity. At first, the nanoparticles were analyzed by UV. The UV–visible spectral analysis of shilajit extract and ZnO nanoparticles from shilajit extract are shown in Fig. 1B,C. ZnO nanoparticles synthesized using shilajit extract displayed absorbance peaks at 357.90 nm and shilajit extract shows absorbance peaks at 422.40 nm. The shift of the absorbance peak towards a higher wavelength indicates the reduction in size from bulk molecules to the nano range. This may be due to material transitions, when an electron obtains energy, it transitions from a lower to a higher energy level^43^.

### FTIR analysis of ZnO NPs

**Figure 2:**
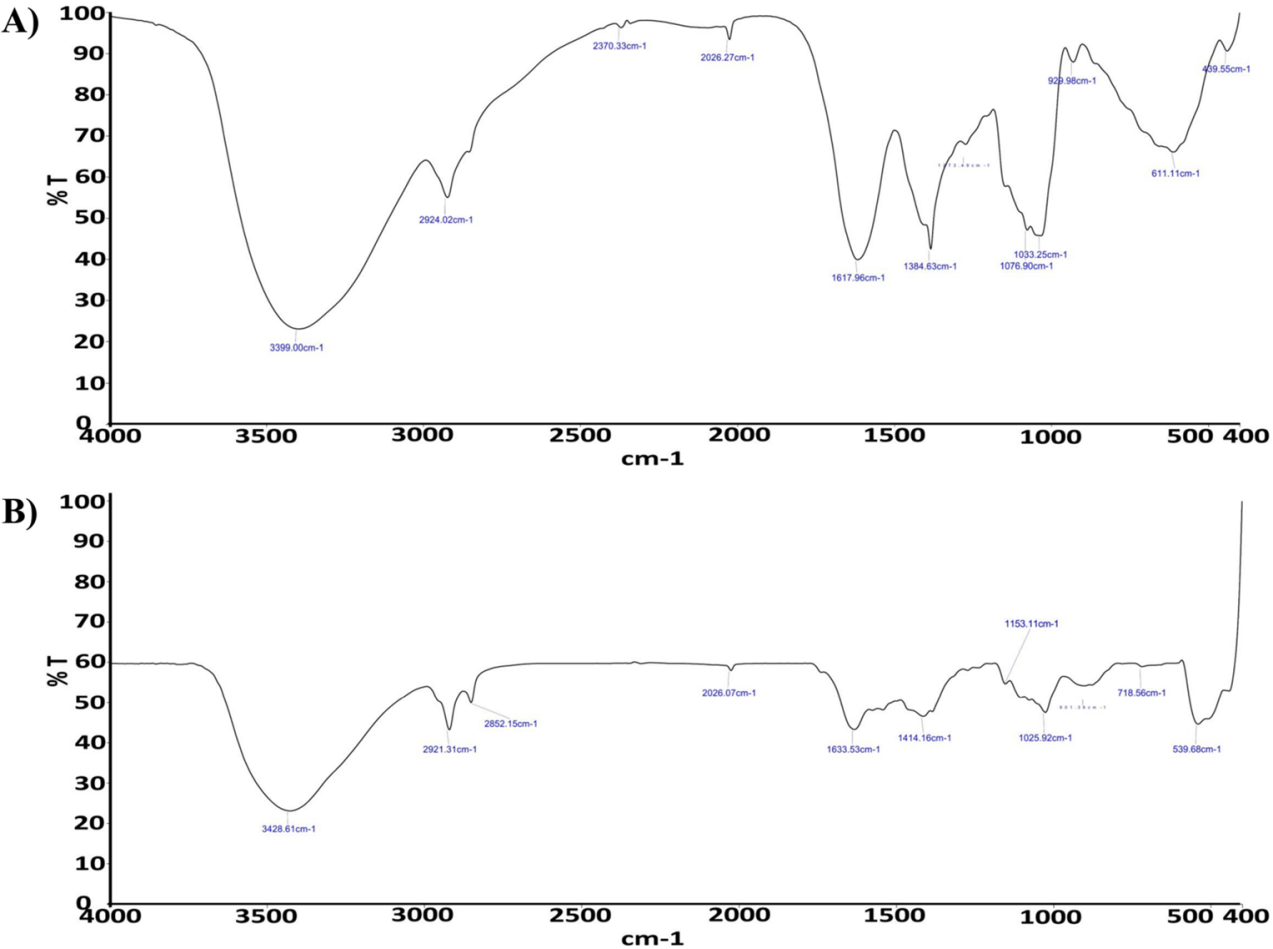
FTIR spectrum of (**A**) shilajit extract and (**B**) ZnO nanoparticles from shilajit extract.

The FTIR spectra of the shilajit extract and the synthesized ZnO nanoparticles from the shilajit extract are shown in Fig. 2. FTIR of the shilajit extract exhibited a broad peak at about 3399 cm^−1^ which can be attributed to the stretching vibration of OH group and a peak at 2924 cm^−1^ confirmed the stretching vibration of aliphatic C–H, the presence of peaks at 1617 cm^−1^, 1384 cm^−1^ confirmed the presence of C=O of carbonyl group and C=C of aromatic system, respectively. C–O stretching was absorbed in 1033 cm^−1^ which is further the zinc oxide nanoparticles synthesized from shilajit exhibited a characteristic peak at 3428 cm^−1^, 2921 cm^−1^, 1633 cm^−1^, 1415 cm^−1^, 1025 cm^−1^ and which was resembles of shilajit extract. The absorption peak associated with the Zn–O stretching band is clearly absorbed at 539 cm^−1^, which confirms the creation of ZnO-NPs. FTIR analysis of zinc nanoparticles revealed the presence of alkanes, phenol, alcohols, aromatics, alkenes, alkyl halides, and aliphatic amines vibrations^44^. Furthermore, –C=O–, C–O–C, and C–O stretching vibrations were shown to generate maxima in carboxylic acid, polysaccharide, and amino acid, respectively^45^.

### XRD analysis of ZnO NPs

**Figure 3:**
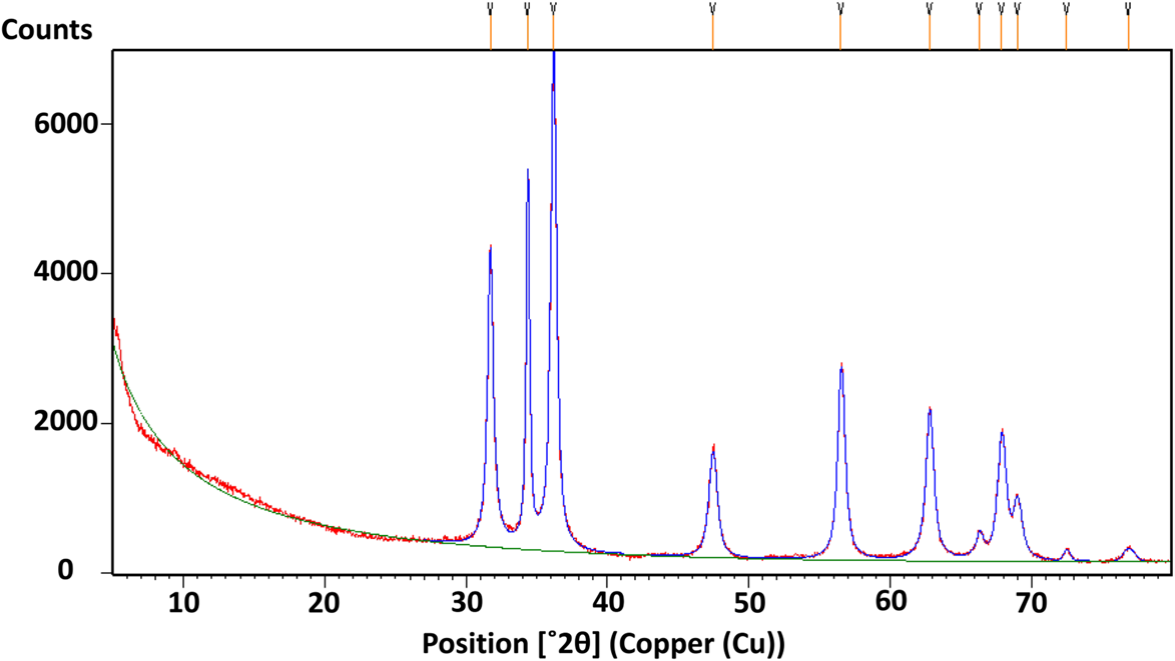
X-ray diffraction pattern of ZnO nanoparticles (synthesized from an aqueous extract of shilajit).

X-ray diffraction (XRD) is a well-established technique used for the structural identification and determination of crystalline size in synthesized ZnO nanoparticles. In this study, the XRD pattern of the synthesized ZnO nanoparticles is presented in Fig. 3. The X-ray diffraction peaks, characterized by their high intensity, were observed at specific 2θ values, namely 31.69°, 34.33°, 36.17°, 47.45°, 56.52°, 62.77°, 67.87°, and 68.99°. These peaks correspond to the lattice planes (100), (002), (101), (102), (110), (103), (112), and (201), respectively. By comparing the observed XRD pattern with the standard JCPDS (Joint Committee on Powder Diffraction Standards) data, it was found that all the peaks matched with the standard JCPDS card no. 76-0704. This confirms that the synthesized ZnO nanoparticles possess a wurtzite hexagonal-type crystal structure. The XRD analysis provides important insights into the crystalline nature and structural characteristics of the synthesized ZnO nanoparticles. The matching of the diffraction peaks with the standard data validates the crystal structure and confirms the successful synthesis of ZnO nanoparticles in the desired hexagonal structure. The crystallite size (D) was calculated using Debye–Scherrer’s formula:

$${\text{D}} = {\text{K}}\uplambda /\upbeta \; {{\cos}}\uptheta$$

where, D—crystalline size of zinc oxide; λ—wavelength of X-ray source 0.15406 nm in (XRD), β—full width at half maximum of the diffraction peak—Scherer, s constant (0.94), θ—Bragg angle.

### Particle size analysis of ZnO NPs

**Figure 4:**
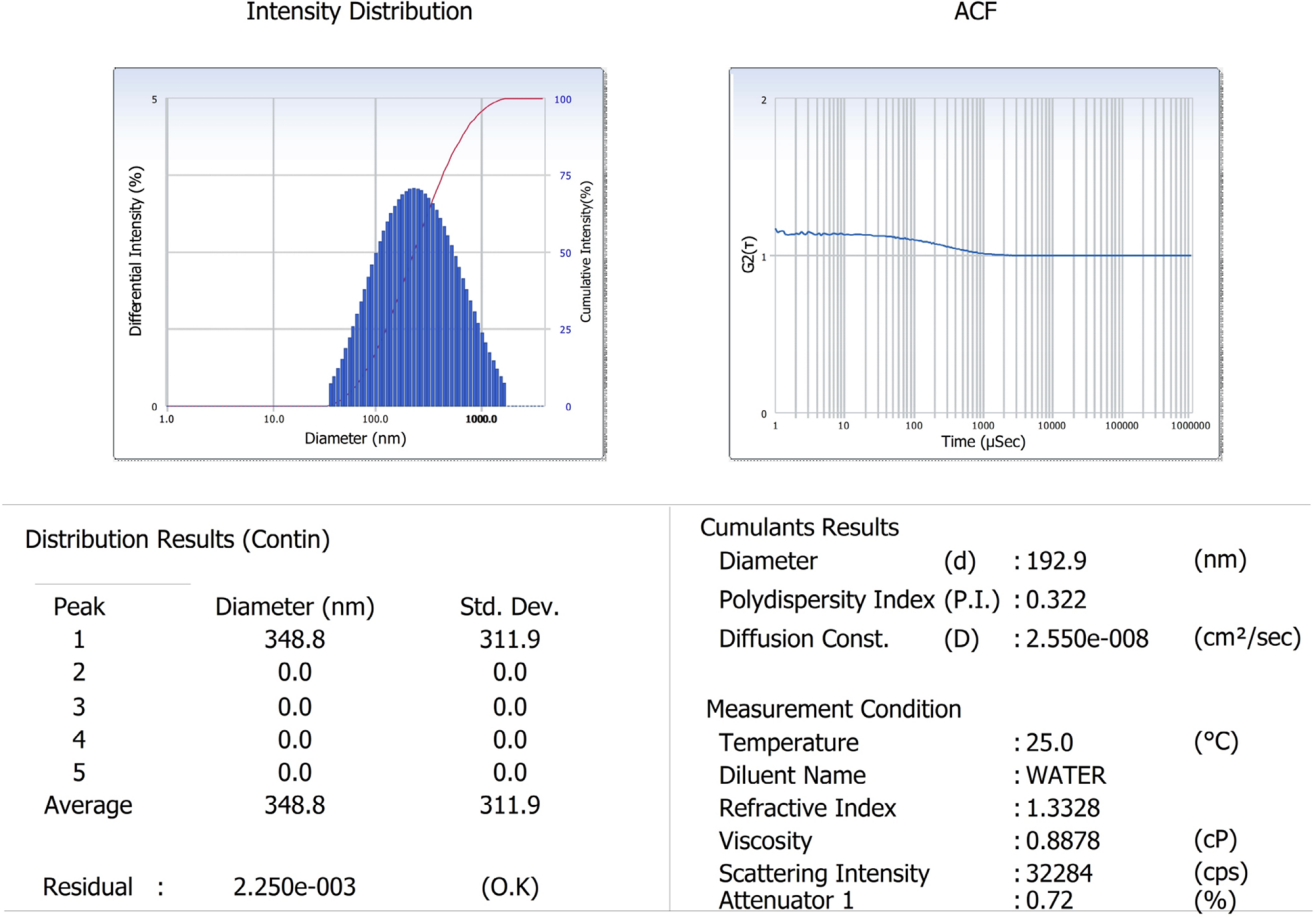
Particle size analysis of ZnO nanoparticles from shilajit extract.

The data obtained from the particle size analyzer provided clear evidence that ZnO nanoparticles synthesized using shilajit extract were smaller. The average size of the nanoparticles obtained with shilajit extract was measured to be 348 nm and the polydispersity index was found to be 0.322 (Fig. 4). These results indicated that the shilajit extract yielded ZnO nanoparticles with a smaller overall size.

### SEM analysis of ZnO NPs

**Figure 5:**
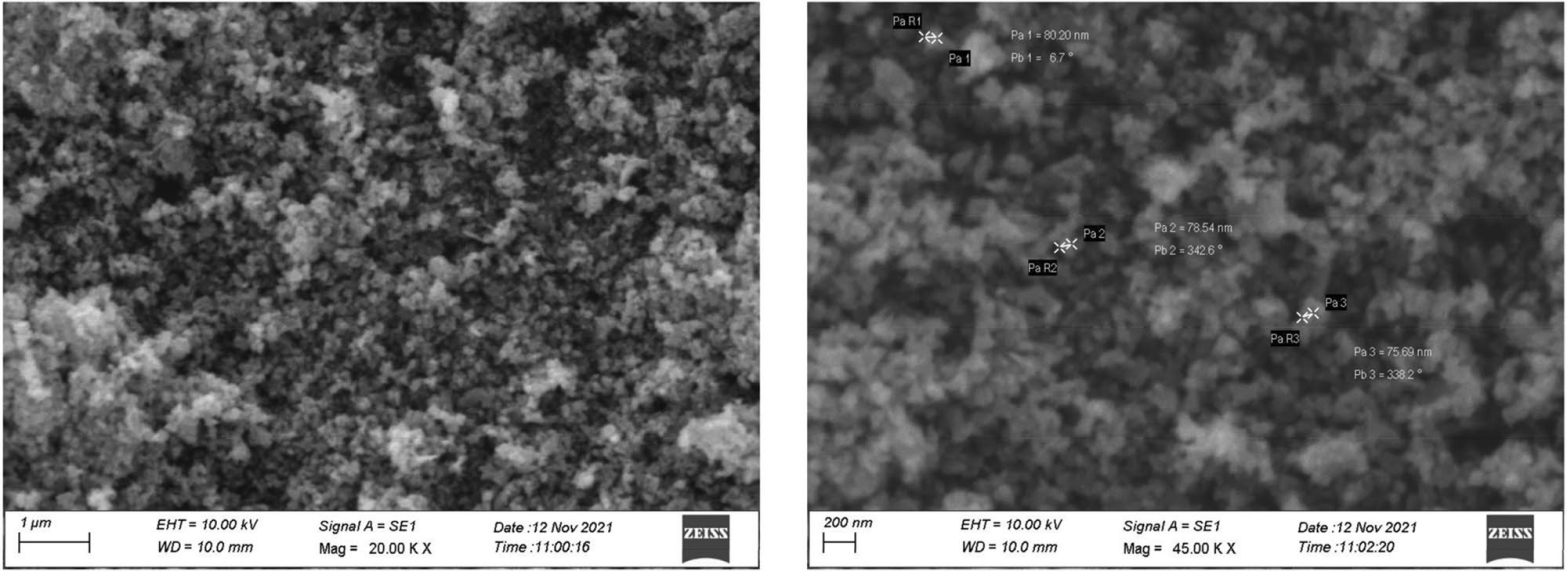
SEM images of ZnO nanoparticles at different magnification from the shilajit extract.

The morphology, including the size and shape, of the green synthesized ZnO nanoparticles was determined through scanning electron microscopy. The SEM images provided insights into the structural characteristics of the synthesized nanoparticles. Specifically, ZnO nanoparticles synthesized from shilajit extract exhibited a morphology resembling nano-flakes with sizes ranging from 75 to 400 nm. These findings highlight the nano-flake-like structures of ZnO nanoparticles with shilajit extract yielding spherical nanoparticles (Fig. 5).

### EDAX analysis of ZnO NPs

**Figure 6:**
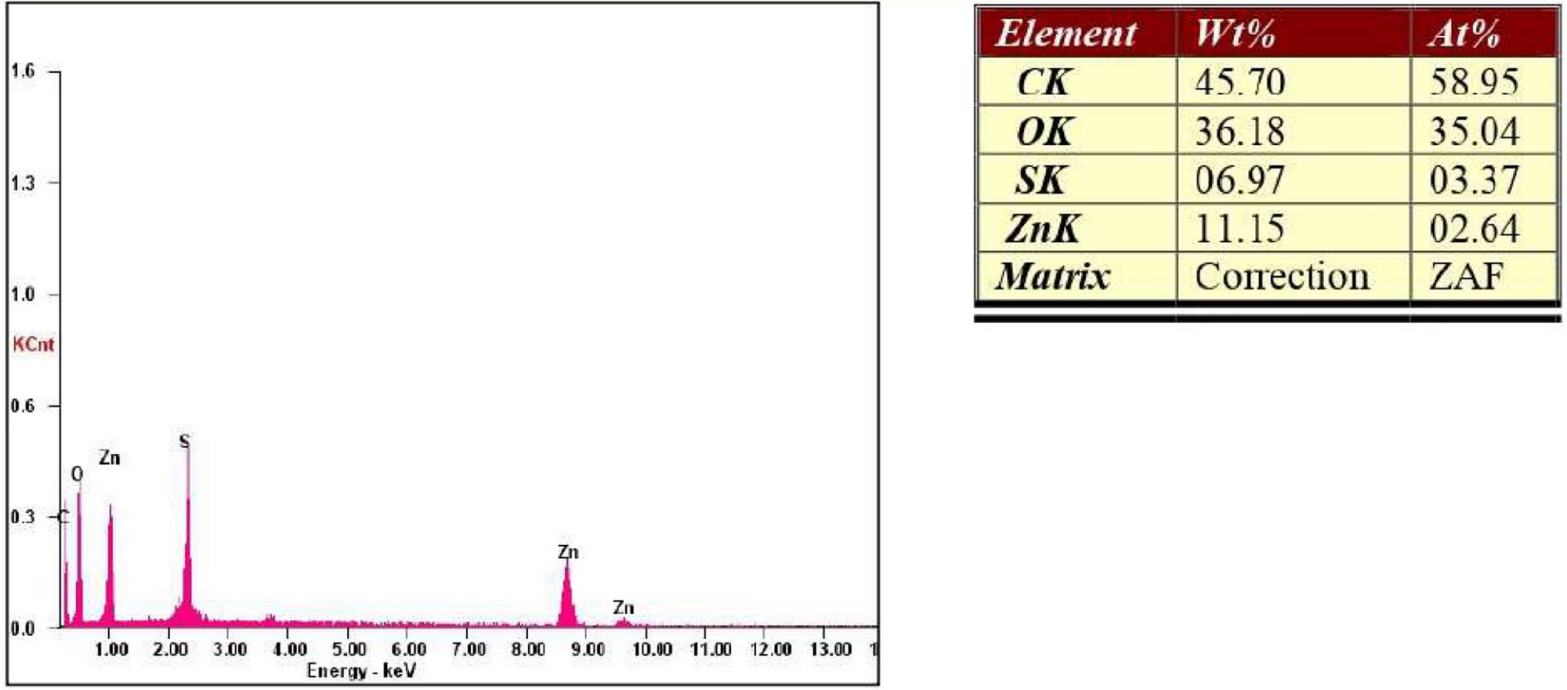
EDAX analysis of ZnO nanoparticles from shilajit extract.

The EDAX spectra of the NPs confirm the purity of the synthesised ZnO NPs and show that the sample contains the necessary phase of Zn and O. The atomic weight of zinc was 02.64%, while its weight present was 11.15%. At the same time, the atomic weight of oxygen was 35.04%, while its weight present was 36.18%, while the other minor constituents present in the ZnO nanoparticles were due to the presence of shilajit extract, as shown in Fig. 6. The EDAX spectra displayed three peaks for zinc around ~ 1 keV, 8.7 keV and 9.6 keV, correspondingly and a singular peak for oxygen at ~ 0.5 keV, which are typical for ZnO NPs^3^.

### In vitro anticancer effect of ZnO NPs

**Figure 7:**
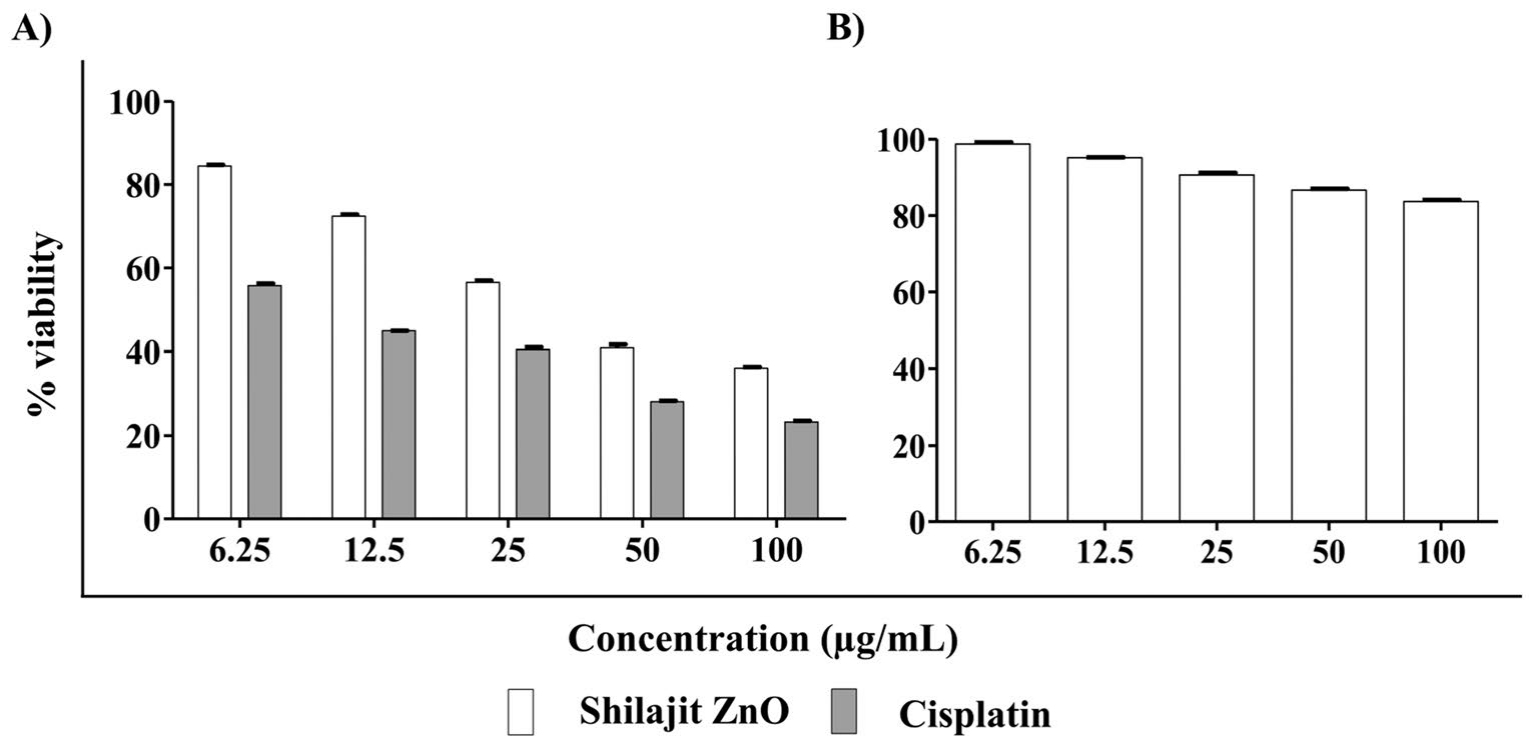
Cytotoxicity assessed by MTT assay after 24 h treatment. (**A**) HeLa cells after treatment with various concentrations (6.25 µg/mL, 12.5 µg/mL, 25 µg/mL, 50 µg/mL and 100 µg/mL) of the ZnO nanoparticles from shilajit extract compared with reference standard cisplatin. (**B**) Normal Vero cells after treatment with various concentrations (6.25 µg/mL, 12.5 µg/mL, 25 µg/mL, 50 µg/mL and 100 µg/mL) of the ZnO nanoparticles from shilajit extract.

**Figure 8:**
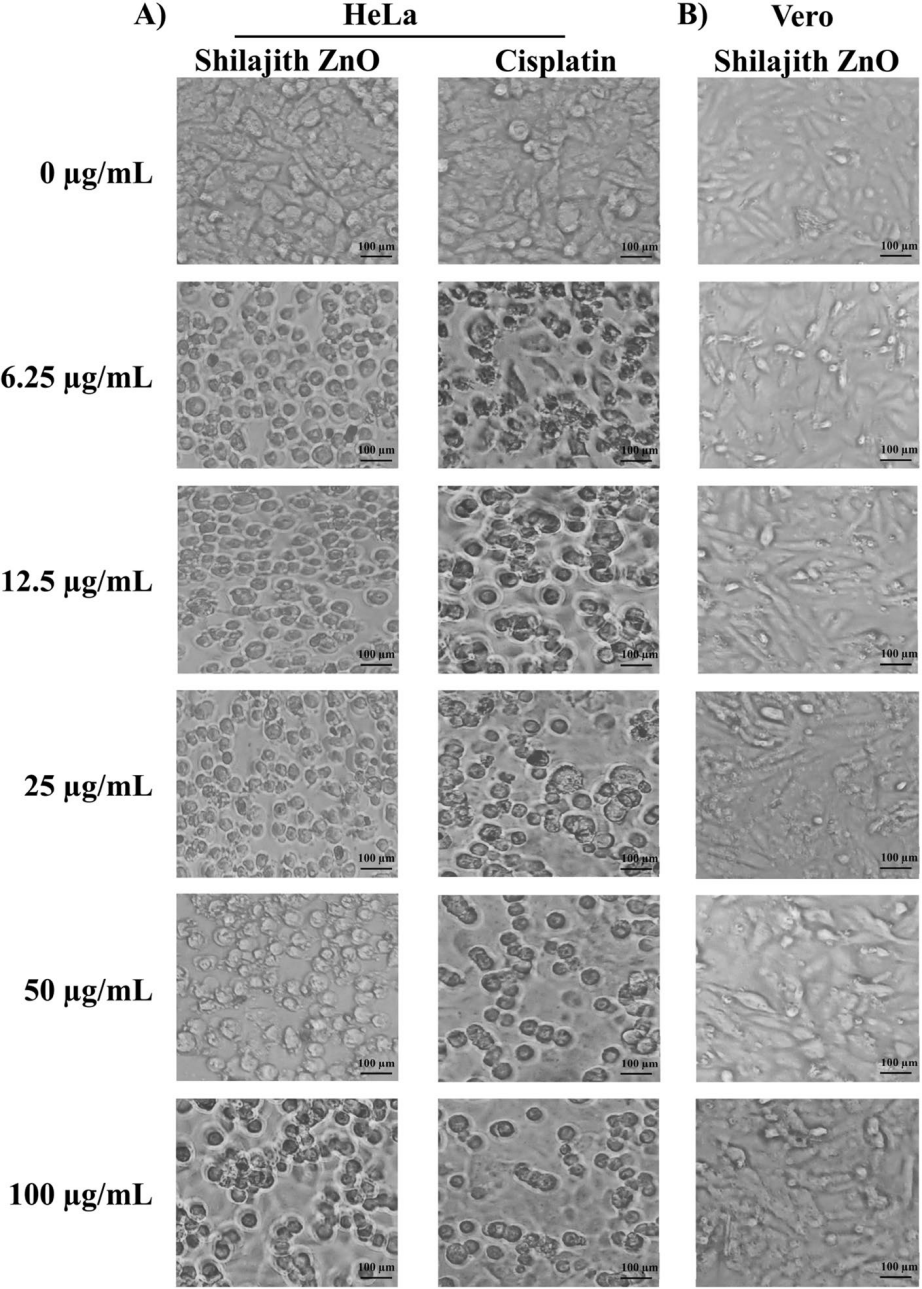
Microscopic images of cells treated with various concentrations after 24 h. (**A**) HeLa cells after treatment with various concentrations (6.25 µg/mL, 12.5 µg/mL, 25 µg/mL, 50 µg/mL and 100 µg/mL) of the ZnO nanoparticles from shilajit extract and reference standard cisplatin. (**B**) Normal Vero cells after treatment with various concentrations (6.25 µg/mL, 12.5 µg/mL, 25 µg/mL, 50 µg/mL and 100 µg/mL) of the ZnO nanoparticles (synthesized from an aqueous extract of shilajit). Cells were observed and photographed by inverted microscopy (20×).

The shilajit extract has been successfully used to synthesize ZnO nanoparticles, which exhibited significant in vitro anticancer activity against human cervical cancer (HeLa) cell lines. The anticancer activity was found to be dose-dependent, with the percentage of viability ranging from 84.47 to 35.98% at concentrations of 6.25 µg/mL to 100 µg/mL, respectively (Figs. 7A, 8A). Notably, the activity of ZnO nanoparticles was comparable to that of cisplatin, a standard anticancer agent, which displayed activity percentages of 55.73% to 23.21% at the same concentrations (Figs. 7A, 8A). Moreover, the IC_50_ value, which represents the concentration at which the ZnO nanoparticles inhibit 50% of cancer cell growth, was determined to be 38.60 µg/mL. This IC_50_ value was moderate to that of the positive control cisplatin, which had an IC_50_ value of 9.43 µg/mL. These results highlighted the potent anticancer activity of the ZnO nanoparticles synthesized using the shilajit extract. The effect of the ZnO nanoparticles on a normal cell line i.e., the Vero cells line was also studied. The MTT assay revealed that the ZnO nanoparticle IC_50_ value was above 100 μg/mL (Figs. 7B, 8B). This data confirmed that the tested ZnO nanoparticles are safe apart from the normal cell line.

**Figure 9:**
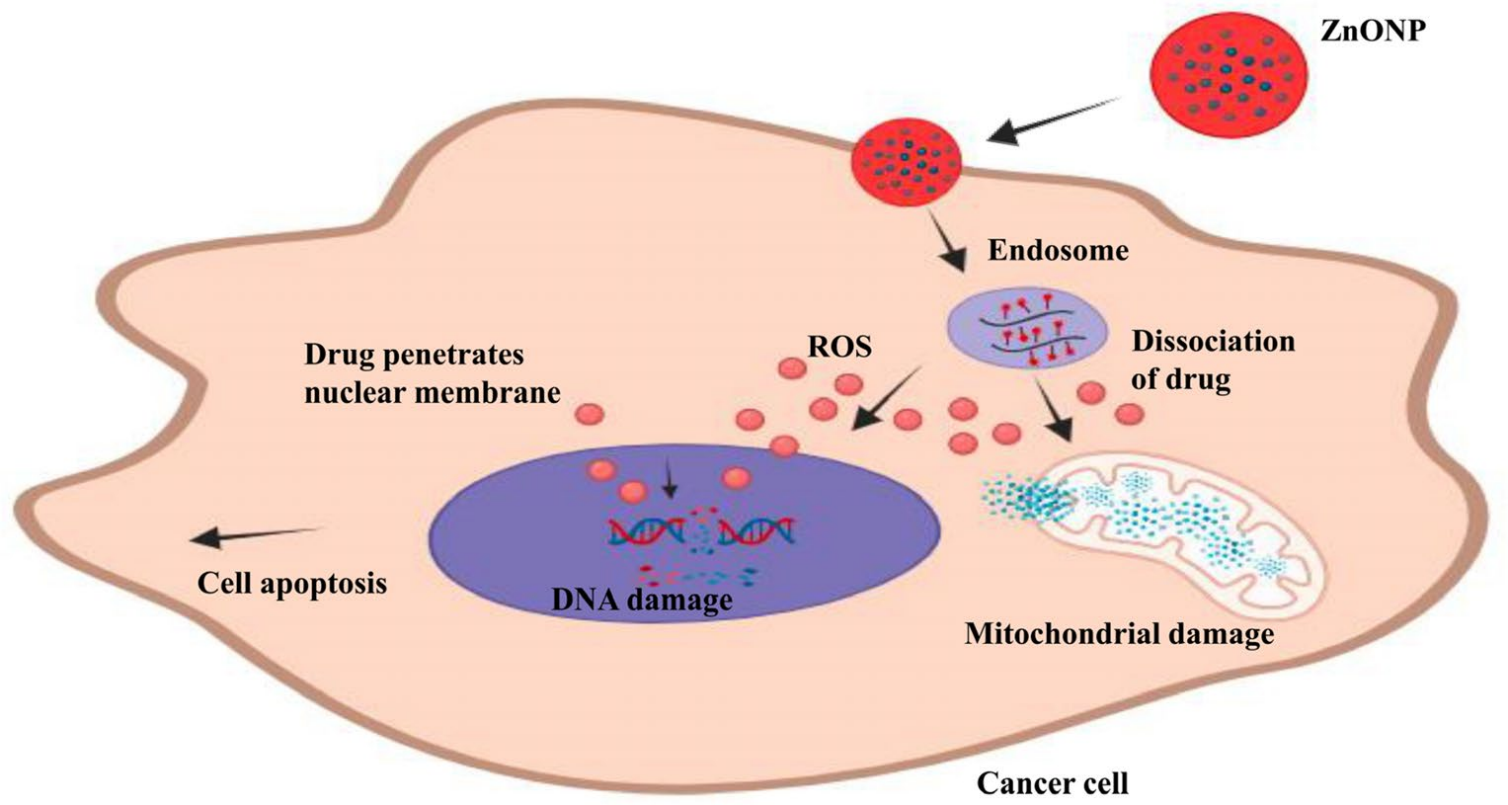
Possible toxic mechanism of zinc oxide nanoparticles against cancer cells.

The anticancer activity of ZnO nanoparticles (ZnONPs) on cancerous cells can be attributed to several potential mechanisms. Firstly, ZnO nanoparticles have been reported to increase the accumulation of intracellular reactive oxygen species (ROS) within cancer cells. ROS are highly reactive molecules that can cause oxidative stress and disrupt cellular processes, leading to DNA damage and ultimately cell death (Fig. 9)^28,46^. Furthermore, ZnO nanoparticles can induce apoptosis, which is a programmed cell death process. Apoptosis is a crucial mechanism for maintaining cellular homeostasis and eliminating damaged or abnormal cells. The stimulation of apoptosis by ZnO nanoparticles suggests that they can trigger signaling pathways that promote cell death in cancer cells^24,25^.

Moreover, ZnO nanoparticles have been shown to induce mitochondrial damage and oxidative stress specifically in cancer cells. Mitochondria play a vital role in regulating various cellular processes, including apoptosis and energy metabolism. By targeting mitochondria, ZnO nanoparticles disrupt the normal functioning of these organelles, leading to impaired energy production and further amplification of oxidative stress within cancer cells. This mitochondrial damage can ultimately trigger cell death pathways specific to cancer cells^26,47^. In summary, the mechanisms underlying the anticancer activity of ZnO nanoparticles involve the induction of intracellular ROS accumulation, stimulation of apoptosis, and disruption of mitochondrial function. These effects collectively contribute to the selective toxicity of ZnO nanoparticles toward cancer cells, making them a promising candidate for cancer therapy^27^.

## Conclusion

The shilajit extract-derived ZnO nanoparticles were studied for their anticancer properties. The high cost and adverse side effects associated with conventional chemotherapeutic agents have necessitated the exploration of natural products as potential alternatives or adjuvants to conventional anticancer drugs. Numerous studies have highlighted the significant potential of shilajit as an anticancer agent. The results demonstrated that zinc oxide nanoparticles of shilajit exhibited promising anticancer properties. However, further research is needed to determine the precise mechanism of action against the cervical cancer, so that this novel ZnO nano-formulation can be verified in a specific in vivo model and further utilized for the good of global society.

## Materials and methods

### Chemicals and reagents

Shilajit was purchased from Yucca Enterprises, India. Zinc acetate was procured from Sisco Research Laboratories Pvt. Ltd. (SRL), India and 0.45 μm filter papers were purchased from Millipore. Isopropanol of analytical grade was purchased from Thermo Fisher Scientific India Pvt. Ltd. All other solvents and chemicals used were of analytical grade. The cisplatin was obtained from the local medical store. Dulbecco’s Modified Eagles Medium (DMEM), Fetal Bovine Serum (FBS), antibiotic and anti-mycotic preparations for cell culture, Trypsin EDTA solution and 3-(4, 5-Dimethylthiazol-2-yl)-2, 5-Diphenyltetrazolium bromide) (MTT) were obtained from Hi Media Laboratories Pvt. Ltd., India.

### Preparation of shilajit extract

About 5 g of shilajit powder was added to 100 mL double distilled water in a 250 mL beaker and heated at 60 °C for 15 min. The mixture was cooled at room temperature and filtered using Whatman No.1 filter paper to obtain a clear solution by removing the solid debris. The filtrate thus obtained was stored at 4 °C for further studies.

### Green synthesis of zinc oxide nanoparticles

Initially, 35 mL of zinc acetate solution (200 mM) was taken in a clean beaker. 15 mL of shilajit extract was added to the zinc acetate solution in a dropwise manner. After addition, the mixture was stirred for 6 h to ensure thorough mixing. After 6 h, sodium hydroxide solution (2 M) was added and kept on a magnetic stirrer at 60 °C overnight. The mixture was allowed to cool down and centrifuged at 12,000 rpm for 15 min. The ZnO nanoparticles thus obtained were washed with double distilled water followed by isopropanol and dried at 50 °C for 2 h (Fig. 1A)^48^.

### Characterization of ZnO NPs

#### UV–Vis spectroscopy analysis

The formation of zinc nanoparticles was confirmed by measuring the λ max with a UV–Visible spectrophotometer (Shimadzu Corporation, Kyoto, Japan).

#### X-ray diffraction (XRD) analysis

XRD measurement was recorded by an X-ray diffractometer (X'Pert PANalytical) instrument operating at a voltage of 40 kV and current of 30 mA with Cu K (α) radiation to determine the crystalline phase and material identification.

#### FTIR analysis

The fine powders of ZnO nanoparticles were analyzed by FTIR spectroscopy ((Shimadzu Corporation, Kyoto, Japan) to determine the functional groups present in the synthesized nanoparticles.

#### SEM analysis

The particle shape and surface characteristics of the freshly prepared nanoparticle formulations were investigated by scanning electron microscope (SEM) Scanning electron microscopy (SEM; JEOL JMS-6390 apparatus) at 25 ± 2 °C.

#### Particle size analysis

A particle size analyzer (Zetasizer Nano ZS, Malvern Instruments Limited, Worcestershire, United Kingdom) was used to determine the particle size distribution and surface charge of the Nanoparticles.

#### EDAX analysis

The elemental analysis of composition was recorded by energy dispersive X-ray spectrometry (SUTW-SAPHIRE Model detector).

### In vitro anticancer activity

#### Cell lines and culture medium

HeLa cell line (cervical cancer cell line) and Vero cell line was procured from NCCS, Pune, India stock cell was cultured in DMEM medium supplemented with 10% inactivated Fetal Bovine Serum (FBS), penicillin (100 IU/mL), streptomycin (100 μg/mL) in a humidified atmosphere of 5% CO_2_ at 37 °C until confluent.

#### MTT assay

The cell viability of ZnO nanoparticles treated cells was measured using the MTT assay as previously described^49^. The monolayer cell culture was trypsinized and the cell count was adjusted to 1.0 × 10^5^ cells/mL using DMEM media containing 10% FBS. To each well of the 96-well microtiter plate, 100 μL of the diluted cell suspension (1 × 10^4^ cells/well) was added. After 24 h, when a partial monolayer was formed, the supernatant was flicked off, washed the monolayer once with medium and 100 μL of different concentrations (6.25, 12.5, 25, 50 and 100 µg/mL) of test samples were added onto the partial monolayer in microtiter plates. The plate was then incubated at 37 °C for 24 h in 5% CO_2_ atmosphere. After incubation, the test solutions in the wells were discarded and 20 μL of MTT (2 mg/1 mL of MTT in PBS) was added to each well. The plate was incubated for 4 h at 37 °C in a 5% CO_2_ atmosphere. The supernatant was removed, 100 μL of DMSO was added, and the plate was gently shaken to solubilize the formed formazan. The absorbance was measured using a microplate reader at a wavelength of 570 nm. The percentage of viability was calculated using the following formula:

$$\% {\text{ viability }} = {\text{ (Sample OD/Control OD) }} \times { 1}00$$

## Data Availability

The datasets generated and analyzed during the current study are available in the Zenodo repository, 10.5281/zenodo.8329357.
